# Impact of partial nephrectomy on kidney function in patients with renal cell carcinoma

**DOI:** 10.1186/1471-2369-15-181

**Published:** 2014-11-19

**Authors:** Chang Seong Kim, Eun Hui Bae, Seong Kwon Ma, Sun-Seog Kweon, Soo Wan Kim

**Affiliations:** Department of Internal Medicine, Chonnam National University Medical School, 42 Jebongro, Gwangju, 501-757 Korea; Department of Preventive Medicine, Chonnam National University Medical School, Gwangju, Korea; Jeonnam Regional Cancer Center, Chonnam National University Hwasun Hospital, Hwasun, Republic of Korea

**Keywords:** Kidney function, Nephrectomy, Renal cell carcinoma

## Abstract

**Background:**

This study aimed to compare the changes in kidney function and the association of tumor size and renal outcomes between patients with renal cell carcinoma (RCC) who underwent radical nephrectomy (RN) and those who underwent partial nephrectomy (PN).

**Methods:**

A retrospective cohort study was conducted for 557 patients with an RCC of ≤7 cm in diameter and normal contralateral kidney function who underwent PN or RN. PN was performed for 218 (39%) patients. Renal outcomes included the incidence of acute kidney injury (AKI), new-onset chronic kidney disease (CKD), and a ≥25% decline in eGFR 1 year after surgery.

**Results:**

Serial changes in eGFR were compared during the 3 years of follow-up. Postoperative eGFR was significantly lower in patients undergoing RN than in those undergoing PN. The incidence of AKI and new-onset CKD was significantly higher in patients after RN (70.1% vs. 24.3%, respectively; *P* <0.001) than after PN (55.7% vs. 6.2%, respectively; *P* <0.001). According to the multivariable logistic regression analysis, RN was an independent risk factor for a ≥25% decline in kidney function after 1 year regardless of the tumor size, even after adjusting for various covariates.

**Conclusions:**

Compared to PN, RN for even a moderate sized RCC leads to an increased incidence of AKI and new-onset CKD, and is a significant risk factor for kidney function decline. Therefore, PN should be considered as the choice of surgical treatment for RCCs that are ≤7 cm in diameter in order to preserve renal function postoperatively.

## Background

Kidney cancer is the 13th most common malignancy worldwide and ranks 3rd among the leading causes of genitourinary cancers in Korea [1]. Renal cell carcinoma (RCC) accounts for approximately 90% of all renal malignancies. Although the incidence of RCC is declining in some European countries in recent years, a worldwide increase in RCC has been observed during the last decade [2].

Recently, the development and widespread use of imaging technologies have led to a decrease in the size and stage migration of newly detected renal cortical tumors. In the last two decades, increased detection of small renal masses has led to a greater utilization of partial nephrectomy (PN). In patients with small renal cortical tumors, radical nephrectomy (RN) is a significantly independent risk factor for the development of chronic kidney disease (CKD) than PN [3, 4]. Preserving the renal parenchyma by PN should be considered in most patients with small renal tumors because the decreased kidney function following RN might increase the risk for cardiovascular events and overall mortality in the long-term [5–7]. Although it has been shown that the development of CKD or decreased kidney function is higher in patients with RCC after RN than PN [8, 9], the renal outcomes between RN and PN are not well-understood in these patients. In addition, researchers of a recent study demonstrated that the incidence of CKD increased as the renal cortical tumor size decreased following RN [10]. However, data on the changes of kidney function after RN or PN in patients with renal tumors >4 cm are currently limited [11, 12].

We hypothesized that RN influences the risk for deleterious renal outcomes for acute or chronic state patients with small and moderate sized RCCs. The aim of this study was to compare the changes in kidney function and various renal outcomes as well as the association of tumor size and renal outcomes between patients with RCC undergoing RN and those undergoing PN. We hope that the findings of this study will help enhance surgical treatment and improve clinical outcomes for patients with RCC.

## Methods

### Study design and patient population

The electronic medical records were reviewed for all adult patients undergoing RN or PN for kidney cancer between December 2003 and December 2012 at the Chonnam National University Hospital. Among the 916 patients identified, we excluded patients with bilateral tumors and benign renal masses (n = 263), end-stage renal disease (patients with a history of hemodialysis, peritoneal dialysis, or kidney transplantation; n = 12), an estimated glomerular filtration rate (eGFR) <15 mL/min/1.73 m^2^ (n = 1), a RCC >7 cm in diameter (n = 82), and those who had a nephrectomy for metastatic RCC (n = 1). A total of 557 patients were included in the final analysis. RN was conducted in patients with a tumor involving a more central position of the kidney, suspected lymph node involvement, and multiple tumors in a single kidney. Patients with a tumor of <7 cm in size underwent a PN when it was technically feasible. Otherwise, the surgical modality was chosen at the discretion of the surgeon. Cases of death were ascertained by data linkage of the national death certificate database of Statistics Korea and the regional cancer registries. The waiver of informed patient consent and approval of the study protocol were obtained by the Institutional Review Board of Chonnam National University Hospital in 2014. This study was conducted according to the principles of the Declaration of Helsinki.

### Data collection and definition

Laboratory data regarding the levels of serum creatinine (SCr), hemoglobin, and urine protein were obtained from the medical records and reviewed by a trained study coordinator. SCr levels were measured at 6 time points (i.e., preoperatively, at 7 days, during discharge, and 3 months, 1 year, and 3 years after nephrectomy).

Tumor stage was reassessed according to the American Joint Committee on Cancer and the International Union for Cancer Control tumor-node-metastasis classification [13]. The Fuhrman grading system was used to measure nuclear grades [14]. To identify the impact of the type of surgery on renal outcomes according to the tumor size, we divided the patients into the following 2 groups: (1) tumor size ≤4 cm and (2) 4 cm < tumor size ≤7 cm.

Acute kidney injury (AKI) was defined according to the Kidney Disease Improving Global Outcomes clinical practice guidelines. According to these guidelines, AKI is present when an abrupt reduction in kidney function results in an absolute increase in the SCr level by ≥0.3 mg/dL within 48 hours, a known or presumed ≥1.5-fold increase in the baseline SCr level within the prior 7 days, or a reduction in urine output (<0.5 mL/kg/h) for 6 hours [15]. We did not consider the urine output criteria because retrospectively collected data can be inaccurate in this regard. AKI was further classified into the following 3 stages according to the severity of kidney injury: AKI stage 1, increase in the SCr level by ≥0.3 mg/dL or 1.5–1.9 times baseline; AKI stage 2, increase in the SCr level by 2.0–2.9 times baseline; and AKI stage 3, increase in the SCr level by ≥4.0 mg/dL or ≥3.0 times baseline or initiation of renal replacement therapy. The patients who met the AKI criteria were further classified into the transient AKI (normalization of SCr level at discharge; SCr level ≤1.3 mg/dL) and the persistent AKI (sustained elevation of SCr level at discharge; SCr level >1.3 mg/dL) groups.

New-onset CKD was defined as a decrease in the eGFR to <60 mL/min/1.73 m^2^ 3 months after nephrectomy in patients with a preoperative eGFR >60 mL/min/1.73 m^2^.

### Assessment of renal function

SCr levels were analyzed by the Jaffe method, which was calibrated by isotope dilution mass spectrometry. The eGFR (units = mL/min/1.73 m^2^) was calculated using the Chronic Kidney Disease Epidemiology Collaboration equation as follows: 141 × minimum (creatinine/κ, 1)^α^ × maximum (creatinine/κ, 1)^-1.209^ × 0.993^age^ × 1.018 (if female) × 1.159 (if black), where κ is 0.7 for women and 0.9 for men, and α is -0.329 for women and -0.411 for men [16].

### Statistical analysis

Continuous variables were presented as mean ± standard deviation and categorical variables were expressed as the number and percentage of patients. Comparative analysis was performed using Student’s *t*-test for continuous variables and Pearson chi-square test for categorical variables. An analysis of covariance and multiple logistic regressions adjusted to age, sex, and proteinuria were performed to evaluate the changes in eGFR and renal outcomes (including AKI, new-onset CKD, and a ≥25% decline in eGFR after 1 year) between RN and PN at various time points. We performed a two-way analysis of variance (ANOVA) with repeated measures to compare the mean changes of eGFR over time between RN and PN according to tumor size. Within-group comparisons for eGFR at 7 days, 3 months, and 3 years were performed using a general linear model-ANOVA followed by a Bonferroni’s correction applied to the post-hoc analysis for multiple comparisons using a pared *t*-test; a *P* value of 0.017 was considered statistically significant. Multivariable logistic regression analyses were performed to identify the independent predictors of adverse renal outcomes according to tumor size after nephrectomy. Age, sex, blood pressure (systolic and diastolic), body mass index, history of smoking, hypertension, and diabetes mellitus, urine protein levels, type of surgical procedure, and the pathologic stage were all included in the multivariable logistic regression analysis. All statistical tests were 2-tailed and *P* <0.05 was considered significant. The analyses were performed using the Statistical Package for Social Sciences software, version 17.0 (SPSS, Chicago, Illinois, USA).

## Results

### Patient characteristics

A total of 557 patients were included in the retrospective analysis. The mean age of the patients was 60.7 years, 393 (70.6%) patients were male, and the mean baseline eGFR was 81.5 ± 16.7 mL/min/1.73 m^2^. RN and PN were performed in 339 (61%) and 218 (39%) patients, respectively. The clinical characteristics of the patients with RCC are listed in Table 1 according to the type of surgery. Compared to patients undergoing RN, those undergoing PN were more likely to have lower systolic and diastolic blood pressures (130 ± 14 mmHg and 82 ± 10 mmHg vs. 133 ± 14 mmHg and 84 ± 10 mmHg, respectively) and higher baseline eGFR (83.9 ± 15.1 mL/min/1.73 m^2^ vs. 80.1 ± 17.6 mL/min/1.73 m^2^, respectively). However, there were no differences between the RN and PN groups for age, sex, body mass index, hemoglobin levels, degree of urine protein, and a history of hypertension, diabetes, coronary artery disease, cerebral vascular disease, and smoking. Apart from the tumor size and pathologic stage, there were no significant differences in pathological characteristics (including tumor location, histological subtypes, and Fuhrman grade) between the RN and PN groups (Table 2). Open surgery was performed more frequently in the PN group compared to the RN group (22.9% vs. 13.6%, respectively; *P* = 0.004). The overall recurrence rate was greater in the RN group than in the PN group (11.2% vs. 3.2%, respectively; *P* = 0.001). However, there was no difference in recurrence rate at the ipsilateral or contralateral kidney between the RN and PN groups (4.1% vs. 1.8%, respectively; *P* = 0.150).

**Table 1:** **Baseline clinical characteristics**

	All patients (n = 557)	RN (n = 339)	PN (n = 218)	***P***value
Age (years)	60.7 ± 12.3	61.0 ± 12.8	60.3 ± 11.4	0.495
Male (%)	393 (70.6)	234 (69.0)	159 (72.9)	0.323
Systolic blood pressure (mmHg)	132 ± 14	133 ± 14	130 ± 14	0.027
Diastolic blood pressure (mmHg)	83 ± 10	84 ± 10	82 ± 10	0.015
Body mass index (kg/m^2^)	24.4 ± 3.1	24.4 ± 3.1	24.3 ± 3.0	0.748
Diabetes (%)	89 (15.9)	55 (16.1)	34 (15.7)	0.885
Hypertension (%)	220 (39.4)	139 (40.8)	81 (37.3)	0.418
Coronary artery disease (%)	25 (4.5)	13 (3.8)	12 (5.5)	0.339
Cerebral vascular disease (%)	15 (2.7)	7 (2.1)	8 (3.7)	0.247
History of Smoking (%)				0.061
Never smoked	342 (61.4)	203 (59.9)	139 (63.8)	0.359
Ex-smoker	104 (18.7)	58 (17.1)	46 (21.1)	0.238
Current smoker	111 (19.9)	78 (23.0)	33 (15.1)	0.023
Hemoglobin (mg/dL)	13.9 ± 1.7	13.8 ± 1.7	14.1 ± 1.5	0.097
Urine protein (grade)^a^				0.124
0	482 (86.7)	286 (84.6)	196 (89.9)	0.073
1-2	51 (9.2)	32 (9.5)	19 (8.8)	0.764
3-4	23 (4.2)	20 (5.9)	3 (1.4)	0.008
Baseline creatinine (mg/dL)	0.95 ± 0.23	0.97 ± 0.25	0.93 ± 0.18	0.021
Baseline eGFR^b^	81.5 ± 16.7	80.1 ± 17.6	83.9 ± 15.1	0.007

^a^Dip-stick test.

^b^eGFR (mL/min/1.73 m^2^) calculated using the Chronic Kidney Disease-Epidemiology Collaboration equation.

Abbreviations: eGFR estimated glomerular filtration rate, RN radical nephrectomy, PN partial nephrectomy.

**Table 2:** **Baseline pathological characteristics**

	All patients (n = 557)	RN (n = 339)	PN (n = 218)	***P***value
**Surgical approach (%)**				0.004
Open	96 (17.2)	46 (13.6)	50 (22.9)	
Laparoscopic	461 (82.8)	293 (86.4)	168 (77.1)	
**Tumor location (%)**				0.925
Right	285 (51.2)	174 (51.3)	111 (50.9)	
Left	272 (48.8)	165 (48.7)	107 (49.1)	
**Tumor size (cm)**	3.4 ± 1.5	4.1 ± 1.4	2.4 ± 1.0	<0.001
≤ 4, No (%)	370 (66.4)	167 (49.3)	203 (93.1)	<0.001
4 < size ≤7, No (%)	187 (33.6)	172 (50.7)	15 (6.9)	<0.001
**Histology (%)**				0.077
Clear cell	439 (79.0)	270 (79.9)	167 (77.5)	
Papillary	47 (8.5)	20 (5.9)	27 (12.4)	
Chromophobe	43 (7.7)	29 (8.6)	14 (6.5)	
Other	27 (4.9)	19 (5.6)	8 (3.7)	
**Fuhrman grade (%)**				0.721
1	68 (12.2)	38 (11.2)	30 (13.8)	
2	358 (64.3)	218 (64.3)	140 (64.2)	
3	109 (19.6)	67 (19.8)	42 (19.3)	
4	12 (2.2)	9 (2.7)	3 (1.4)	
**Pathologic stage (%)** ^**a**^				0.005
I	546 (98.0)	328 (96.8)	218 (100)	
III	11 (2.0)	11 (3.2)		
**Nephrectomy size (cm)**	9.2 ± 4.7	12.5 ± 2.2	3.9 ± 1.5	<0.001

^a^Based on the tumor node metastasis staging system supported by both the American Joint Committee on Cancer and the International Union for Cancer Control.

Abbreviations: RN radical nephrectomy, PN partial nephrectomy.

### Changes of renal function after PN or RN

**Figure 1:**
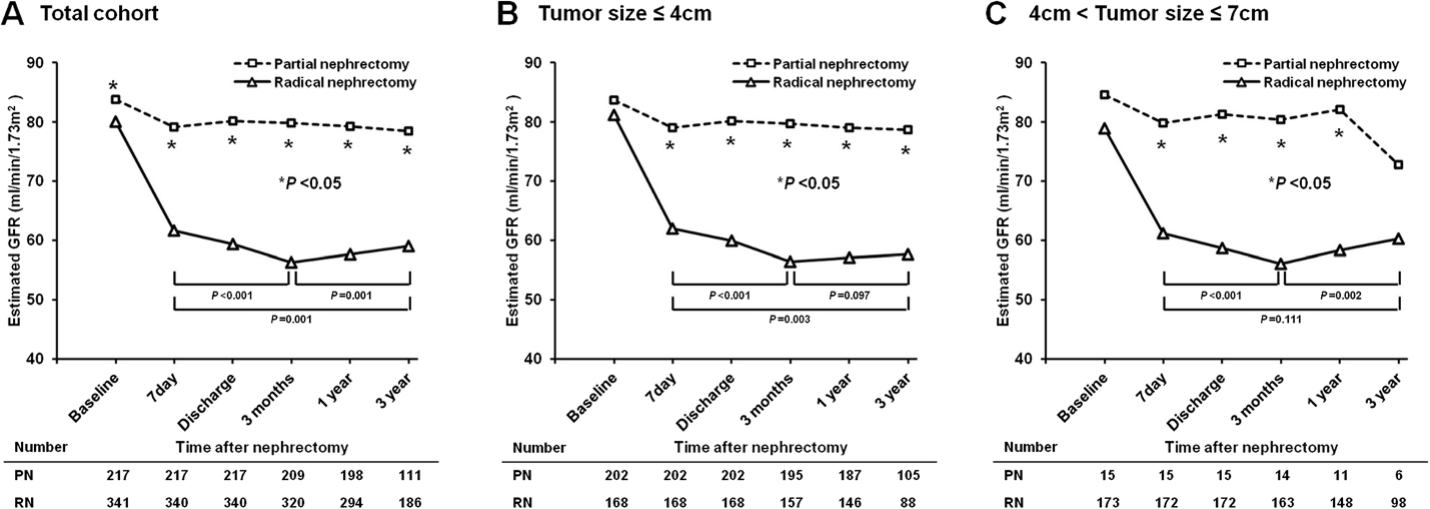
**Serial changes in estimated glomerular filtration rate (eGFR) before and after surgery in patients undergoing radical and partial nephrectomy in the total cohort (A), and patients with a tumor ≤4 cm (B) or 4–7 cm (C) in size.** *P <0.05; vs. eGFR in the radical nephrectomy group at each time point.

Serial changes in the mean eGFR from before surgery to the time points following surgery (i.e., 7 days, at discharge, 3 months, 1 year, and 3 years) according to the type of surgery (i.e., PN and RN) and tumor size (≤4 cm and 4 cm < tumor size ≤7 cm) are shown in Figure 1. The eGFR was significantly lower in patients undergoing RN than in those undergoing PN 3 years after surgery, even after adjusting for age and sex (*P* <0.05), as determined using two-way ANOVA with repeated measure analysis. The RN and PN groups, categorized according to tumor size, also showed similar changes in eGFR after surgery, even though the baseline eGFR was not different between the 2 groups. The mean eGFR decreased from 80.1 mL/min/1.73 m^2^ before surgery to 56.2 mL/min/1.73 m^2^ at 3 months (*P* <0.001) and then increased to 59.0 mL/min/1.73 m^2^ 3 years after RN (*P* = 0.001). However, there were no significant changes in eGFR over time (i.e., from surgery to follow-up period) in patients with PN.

### Renal outcomes after PN or RN

As shown in Table 3, the incidence of AKI in patients undergoing RN was significantly higher than patients undergoing PN (70.1% vs. 24.3%, respectively; *P* <0.001). Furthermore, the incidence of transient and persistent AKI was significantly higher in patients undergoing RN than PN. In addition, compared to the PN group, the RN group had significantly higher prevalence of new-onset CKD 3 months after the operation (55.7% vs. 6.2%, respectively; *P* <0.001) and a ≥25% decline in the eGFR after 1 year (65.4% vs. 8.1%, respectively; *P* <0.001), even after adjusting for age, sex, and proteinuria. All-cause mortality was significantly higher in patients undergoing RN than in those undergoing PN (10.0% vs. 3.7%; *P* = 0.006). Among these patients, there was 1 case of death in a patient undergoing RN, who died due to end-stage renal disease; no cases of deaths were observed in the PN group.

**Table 3:** **Renal outcomes after radical and partial nephrectomy adjusted for age, sex, and proteinuria**

	All patients (n = 557)	RN (n = 339)	PN (n = 218)	***P***value^a^
AKI (%)^b^	290 (52.2)	237 (70.1)	53 (24.3)	<0.001
Stage 1	265 (47.7)	217 (91.6)	48 (90.1)	<0.001
Stage 2	19 (3.4)	16 (6.7)	3 (5.6)	0.033
Stage 3	6 (1.1)	4 (1.7)	2 (3.8)	1.000
Transient AKI (%)	173 (31.1)	124 (36.7)	49 (22.5)	<0.001
Persistent AKI (%)	117 (21.0)	113 (33.4)	4 (1.8)	<0.001
New-onset CKD (%)^c^	190 (36.0)	177 (55.7)	13 (6.2)	<0.001
≥25% decline in eGFR 1 year after surgery (%)^d^	207 (42.2)	191 (65.4)	16 (8.1)	<0.001
All-cause mortality	42 (7.5)	34 (10.0)	8 (3.7)	0.006

^a^ *P* value by age, sex and proteinruia-adjusted logistic regression.

^b^Defined by Kidney Disease: Improving Global Outcomes guideline.

^c^eGFR <60 mL/min/1.73 m^2^ after 3 months postoperatively, calculated using the Chronic Kidney Disease-Epidemiology Collaboration equation.

^d^eGFR (mL/min/1.73 m^2^), calculated using the Chronic Kidney Disease-Epidemiology Collaboration equation.

Abbreviations: eGFR estimated glomerular filtration rate, AKI acute kidney injury, CKD chronic kidney disease, RN radical nephrectomy, PN partial nephrectomy.

We identified independent risk factors for the adverse renal outcomes that were categorized according to the tumor size after nephrectomy by using multivariable logistic regression analysis (Table 4). Among the risk contributors in the total cohort (tumor size ≤7 cm), RN was found to be the most important factor for the incidence of AKI, new-onset CKD, and a ≥25% decline in eGFR after 1 year. Furthermore, compared to patients undergoing PN, those undergoing RN for tumors ≤4 cm and 4–7 cm in size also had a higher risk of a ≥25% decline in eGFR after 1 year after multiple adjustments (odds ratio [OR] = 28.3, 95% confidence interval [CI] = 14.3 56.0, *P* <0.001; OR = 12.6; 95% CI = 1.45–108.9, *P* = 0.022, respectively).

**Table 4:** **Multivariable logistic regression analysis of renal outcomes categorized by tumor size after nephrectomy**

	Total cohort (n = 557)		Tumor size ≤4 cm (n = 370)		4 cm < tumor size ≤7 cm (n = 187)	
	OR (CI 95%)	***P***value	OR (CI 95%)	***P***value	OR (CI 95%)	***P***value
**AKI**						
Age	0.99 (0.97-1.00)	0.149	0.99 (0.97-1.01)	0.398	0.98 (0.96-1.01)	0.210
Male	3.15 (1.92-5.17)	<0.001	3.58 (1.86-6.91)	<0.001	2.24 (1.13-5.26)	0.023
BMI	1.11 (1.04-1.19)	0.002	1.09 (1.00-1.19)	0.054	1.14 (1.02-1.27)	0.019
Smoking	1.01 (0.64-1.59)	0.976	0.80 (0.45-1.40)	0.429	1.56 (0.69-3.50)	0.283
Hypertension	1.39 (0.89-2.18)	0.144	1.38 (0.78-2.45)	0.275	1.43 (0.69-2.97)	0.338
Diabetes	0.68 (0.38-1.21)	0.188	0.56 (0.27-1.17)	0.122	0.98 (0.36-2.70)	0.967
Proteinuria	1.00 (0.54-1.85)	0.994	1.08 (0.49-0.34)	0.859	0.87 (0.31-2.48)	0.796
Open surgery	1.06 (0.63-1.79)	0.829	1.04 (0.54-2.01)	0.901	1.15 (0.46-2.85)	0.770
Stage III	0.69 (0.18-2.58)	0.576	0.43 (0.02-8.43)	0.575	0.93 (0.19-4.67)	0.931
Partial nephrectomy	1 [Reference]		1 [Reference]		1 [Reference]	
Radical nephrectomy	9.57(6.20-14.8)	<0.001	12.8 (7.09-20.7)	<0.001	5.49 (1.52-19.9)	0.009
**New-onset CKD**						
Age	1.06 (1.04-1.08)	<0.001	1.06 (1.03-1.09)	<0.001	1.06 (1.03-1.10)	<0.001
Male	1.04 (0.60-1.78)	0.898	0.99 (0.46-2.13)	0.988	1.01(0.45-2.24)	0.988
BMI	1.00 (0.92-1.07)	0.884	0.99 (0.89-1.10)	0.879	0.97 (0.87-1.08)	0.580
Smoking	1.49 (-0.89-2.51)	0.131	1.62 (0.80-3.25)	0.178	1.60 (0.70-3.67)	0.266
Hypertension	0.87 (0.54-1.41)	0.564	0.98 (0.50-1.90)	0.943	0.70 (0.33-1.47)	0.345
Diabetes	1.52 (0.81-2.84)	0.190	0.97 (0.42-2.23)	0.940	3.38 (1.13-10.1)	0.030
Proteinuria	0.93 (0.48-1.78)	0.815	1.08 (0.46-2.52)	0.859	0.81 (0.26-2.54)	0.722
Open surgery	0.95 (0.49-1.83)	0.874	1.31 (0.54-3.18)	0.555	0.57 (0.21-1.54)	0.266
Stage III	0.44 (0.11-1.84)	0.262	1.36 (0.08-24.1)	0.836	0.27( 0.05-1.48)	0.131
Partial nephrectomy	1 [Reference]		1 [Reference]		1 [Reference]	
Radical nephrectomy	23.7(11.7-41.0)	<0.001	24.9 (11.7-49.7)	<0.001	7.03 (1.18-31.3)	0.031
**≥25% decline in eGFR 1 year after surgery**						
Age	1.01 (0.99-1.03)	0.309	1.01 (0.99-1.04)	0.371	1.01 (0.98-1.04)	0.655
Male	1.58 (0.91-2.74)	0.104	1.99 (0.92-4.29)	0.081	1.03 (0.45-2.38)	0.944
BMI	1.05 (0.97-1.13)	0.220	1.02 (0.92-1.13)	0.712	1.06 (0.94-1.19)	0.362
Smoking	1.10 (0.65-1.83)	0.731	0.77 (0.38-1.55)	0.469	1.85 (0.89-4.33)	0.158
Hypertension	0.85 (0.51-1.41)	0.527	0.68 (0.35-1.36)	0.277	1.07 (0.48-2.40)	0.868
Diabetes	1.57 (0.81-3.03)	0.178	1.64 (0.69-3.86)	0.261	1.50 (0.50-4.49)	0.472
Proteinuria	0.99 (0.50-1.96)	0.971	1.24 (0.50-3.07)	0.638	0.95 (0.31-2.95)	0.930
Open surgery	0.57 (0.29-1.12)	0.102	1.12 (0.46-2.74)	0.805	0.24 (0.08-0.73)	0.011
Stage III	0.77 (0.16-3.71)	0.742	0.55 (0.03-10.5)	0.690	0.69 (0.09-5.10)	0.712
Partial nephrectomy	1 [Reference]		1 [Reference]		1 [Reference]	
Radical nephrectomy	22.7 (12.7-40.7)	<0.001	28.3 (14.3-56.0)	<0.001	12.6 (1.45-108.9)	0.022

Adjusted factors include age, sex, systolic blood pressure, diastolic blood pressure, body mass index, history of smoking, hypertension, and diabetes mellitus, urine protein level, type of surgical procedure, and pathologic stage.

Abbreviations: OR odds ratio, CI confidential interval, AKI acute kidney injury, CKD chronic kidney disease, eGFR estimated glomerular filtration rate, BMI body mass index.

## Discussion

In this study, we determined that the postoperative eGFR was significantly lower in patients undergoing RN than those undergoing PN during the 3 years follow-up period. Patients undergoing RN also had a higher risk of various adverse renal outcomes (i.e., AKI, new-onset CKD, and ≥25% decline in eGFR after 1 year) compared to those undergoing PN. Furthermore, RN increased the risk for deleterious renal outcomes for patients with small (tumor size ≤4 cm) and moderately (4 cm < tumor size ≤7 cm) sized RCCs. Therefore, our hypothesis that RN increases the risk for deleterious renal outcomes for acute or chronic state patients with small and moderately sized RCCs is supported by the findings of the present study.

RN is now recognized as an independent risk factor for the development of CKD when used as a treatment for small renal tumors [8, 10]. Nevertheless, RN remained the most common treatment for newly detected small renal tumors over the last decade. A recent meta-analysis showed that PN confers a survival advantage and a lower risk of severe CKD (eGFR <60 mL/min/1.73 m^2^) after operation for localized renal tumors [17]. Although RN accounts for up to 60% of all nephrectomies in our cohort, we also found that the incidence of new-onset CKD was higher in patients undergoing RN than those who were undergoing PN (55.7% vs. 6.2%, respectively). However, most studies have investigated the development of CKD at irregular time points after nephrectomy, and there are limited data regarding AKI or kidney function decline after operation [3–5, 8, 11, 18]. We showed that the incidence of AKI was higher in the RN group compared to the PN group. Interestingly, persistence AKI at discharge was high as 33.4% in patients after RN, while it was as low as 1.8% in patients after PN. These findings are in accordance with another study, which conclude that postoperative AKI in patients with RCC is associated with new-onset CKD after RN [19]. Researchers of recent studies have showed that AKI increases the risk of CKD and end-stage renal disease [20–22]. Furthermore, patients who underwent RN had a higher risk of ≥25% decline in kidney function after 1 year than those who underwent PN. Consequently, RN might have influenced the postoperative acute and long-term kidney injury in patients with RCC. According to these changes, PN would be the preferred surgical treatment modality for treating RCC rather than RN because of the advantage of preserving renal function after the operation [23]. The cancer-specific survival for patients with small renal cortical tumors is >90% across all histological subtypes and therefore, there is a need for long-term management of postoperative complication (i.e., decreased kidney function) [24].

Complete surgical excision by PN is recommended for patients with all clinical T1 kidney tumors (i.e., tumor size <7 cm) based on compelling data demonstrating that RN is associated with an increased risk of CKD [25, 26]. Interestingly, it has been reported that as the tumor size decreased, the risk of new-onset CKD increased in patients undergoing RN for RCC [10]. However, only a few studies have been published regarding the link between large tumor sizes and decreased kidney function after RN or PN. In a few retrospective studies, RN was found to be associated with the development of CKD in patients treated for renal tumors of 4–7 cm in size [11, 12, 27]. In this regard, our findings are consistent with the idea that compared to PN, RN is associated with AKI, kidney function decline, and new-onset CKD in patients with moderate sized renal tumors (i.e., 4 cm ≤ tumor size <7 cm). If technically feasible, PN might enable a better preservation of postoperative kidney function and also prevent the development of AKI or CKD for patients with tumors that are up to 7 cm in size.

A lower eGFR before surgery would affect the postoperative kidney function. Although the preoperative eGFR was significantly lower in patients undergoing RN than in those undergoing PN in the total cohort, which might be due to the fact that RN was performed more frequently in patients with RCC >4 cm in size, there was a correlation between increased tumor size and decreased preoperative eGFR [10]. However, there are no significant differences in preoperative eGFR between the patients in the RN and PN groups according to tumor size. Therefore, the effect of tumor size on preoperative eGFR would be attenuated when patients were categorized into the 2 groups by a tumor size >4 cm. Nevertheless, the RN group showed a greater decline in eGFR than the PN group during the 3-year postoperative follow-up period.

According to two retrospective studies, compensatory hypertrophy in the non-operated healthy kidney occurs between 7 days and 4 weeks after RN [10, 28]. However, unlike previous reports, we showed that the non-operated healthy kidney experiences an adaptation in renal function 3 months after RN. Therefore, we should monitor renal function until at least 3 months after RN to prevent further renal injury. In addition, a higher resistive index on duplex ultrasonography, which was not performed in this study, may be helpful for predicting AKI and CKD progression after surgery [29].

The present study has several limitations. Firstly, our study was of retrospective nature and therefore, our findings may be affected by confounding factors or selection bias. Secondly, we could not evaluate the non-neoplastic kidney disease, which may have had an effect on kidney function decline in patients with RCC after nephrectomy. However, there were no differences in comorbidities (i.e., prevalence of hypertension and diabetes that could lead to parenchyma kidney diseases) between patients undergoing RN and PN in this study. Thirdly, long-term renal outcomes could not be fully evaluated despite checking the eGFR at various time points after the operations.

Despite these limitations, our study had some strengths. Firstly, we used the recently validated Chronic Kidney Disease Epidemiology Collaboration equation to calculate the eGFR, while most previous studies involved the Modification of Diet in Renal Disease equation that systematically underestimates GFR in individuals without known CKD [30]. Secondly, we evaluated various renal outcomes including AKI, CKD, and kidney function decline to determine the clinical impact of the surgical procedures. Finally, we collected data regarding the degree of proteinuria in our patients and included these in our analyses for adjustment, which may affect overall kidney function.

## Conclusions

The risk for the incidence of AKI, new-onset CKD, and kidney function decline in patients undergoing RN was higher than that of patients undergoing PN even for moderately sized RCC tumors. Therefore, PN should be considered as the surgical treatment of choice for RCCs up to 7 cm in size, which will allow for the preservation of renal function postoperatively.

## Abbreviations

RCC: Renal cell carcinoma
PN: Partial nephrectomy
RN: Radical nephrectomy
CKD: Chronic kidney disease
eGFR: Estimated glomerular filtration rate
SCr: Serum creatinine
AKI: Acute kidney injury
ANOVA: Analysis of variance.
